# Use of Fermented Red Clover Isoflavones in the Treatment of Overactive Bladder in Postmenopausal Women: A Randomized, Double-Blinded, Placebo-Controlled Trial

**DOI:** 10.3390/nu15194165

**Published:** 2023-09-27

**Authors:** Annemarie B. Villadsen, Julie N. Holm-Jacobsen, Bala K. Prabhala, Caspar Bundgaard-Nielsen, Pam Huntjens, Jette B. Kornum, Karin Glavind, Peter D. C. Leutscher, Lars P. Christensen, Per B. Jeppesen, Suzette Sørensen, Louise T. S. Arenholt

**Affiliations:** 1 Centre for Clinical Research, North Denmark Regional Hospital, 9800 Hjoerring, Denmark; annemarie.j@rn.dk (A.B.V.); jnhj@hst.aau.dk (J.N.H.-J.); c.bundgaardnielsen@rn.dk (C.B.-N.); p.huntjens@rn.dk (P.H.); p.leutscher@rn.dk (P.D.C.L.); suzette.soerensen@rn.dk (S.S.); 2 Department of Clinical Medicine, Aalborg University, 9000 Aalborg, Denmark; 3 Department of Physics, Chemistry and Pharmacy, University of Southern Denmark, 5230 Odense M, Denmark; bapra@sdu.dk (B.K.P.); lpc@sdu.dk (L.P.C.); 4 Department of Clinical Microbiology, Aalborg University, 9000 Aalborg, Denmark; j.kornum@rn.dk; 5 Department of Obstetrics and Gynecology, Aalborg University Hospital, 9000 Aalborg, Denmark; kagl@rn.dk; 6 Department of Clinical Medicine, Aarhus University, Aarhus University Hospital, 8200 Aarhus N, Denmark; per.bendix.jeppesen@clin.au.dk; 7 Steno Diabetes Center North Denmark, 9000 Aalborg, Denmark; 8 Department of Obstetrics and Gynecology, North Denmark Regional Hospital, 9800 Hjoerring, Denmark

**Keywords:** overactive bladder, isoflavones, red clover, urinary incontinence, postmenopause

## Abstract

Postmenopausal women are at risk of developing an overactive bladder (OAB). Conventional vaginal estrogen has shown promise for symptom relief. Isoflavones have proven effective as an alternative to estrogen treatment against menopause-related symptoms. However, its effect on OAB symptoms has not been studied. This study investigates if fermented red clover isoflavones reduce OAB symptoms in postmenopausal women. In this randomized, double-blinded, placebo-controlled trial, women were administered red clover extract (RCE) or a placebo twice daily for three months. Women filled out the International Consultation on Incontinence Questionnaire Overactive Bladder (ICIQ-OAB) and Urinary Incontinence Short Form (ICIQ-UI-SF), together with a fluid intake and voiding diary. A total of 33 women (16 in the RCE group and 17 in the placebo group) were included in the analysis. Baseline demographics and OAB characteristics were comparable across groups. Intake of RCE did not lead to significant relief in most urinary bladder symptom measures, although a significant reduction in the bother of urinary urgency (*p* = 0.033) and a tendency towards a decreased ICIQ-OAB score were observed (*p* = 0.056). In contrast, the placebo exhibited a significant decrease in the ICIQ-OAB score (*p* = 0.021) and in some diary outcomes. We found that an intake of isoflavones did not relieve OAB symptoms in postmenopausal women.

## 1. Introduction

Overactive bladder (OAB) is a common medical condition that may significantly reduce quality of life [1]. It is defined by symptoms of “urinary urgency, usually accompanied by frequency and nocturia, with or without urgency incontinence, in the absence of urinary tract infection or other obvious pathology” [2,3]. The prevalence of OAB is 13–17% worldwide and increases with age [4,5,6]. Changes in hormonal levels during menopause, in particular the decline in estrogen, have been shown to affect the female urinary tract and are believed to play a role in the development of OAB [7]. Consequently, hormonal replacement therapy has been widely used to treat lower urinary tract symptoms in postmenopausal women and might, in theory, help reverse symptoms related to postmenopausal estrogen decline. However, treatment of OAB symptoms with exogenous estrogen has been debated in recent years due to conflicting results from previous trials [7,8,9]. Most clinical studies report a beneficial effect of local vaginal estrogen for treatment of OAB symptoms [10,11,12,13,14], whereas no improvement has been observed by systemic estrogen therapy [15,16,17,18,19]. 

Isoflavones, a class of phytoestrogens, are estrogen-like compounds naturally occurring in legumes such as soybeans and red clover. The primary isoflavones in soybeans are daidzein and genistein, while formononetin and biochanin A are the major isoflavones found in red clover [20]. Various isoflavones have different bioactivity, and the formulation of the isoflavones may influence their properties. It has been found that isoflavone preparations, rich in aglycones, have higher bioavailability [21,22]. Whereas estrogen acts as an agonist with binding affinity towards both estrogen receptor alpha (ER-α) and beta (ER-β), isoflavones are selective against ER-β and show agonistic and antagonistic activities towards these receptors [23,24]. Thus, the physiologic effect may be distinct from that of estrogen. Notably, both ER-α and ER-β are present in the lower urinary tract, including the urethra, vagina, and bladder [25,26,27,28,29,30,31,32,33,34,35,36]. Isoflavones are widely tested as an alternative to estrogen treatment of postmenopausal symptoms and have proven effective against climacteric symptoms [37,38,39,40] and estrogen-deficient bone mineral density loss [41,42]. Furthermore, isoflavone supplementation has shown no increased risk of breast cancer or cancer of the uterus [43]. 

Evidence of the effect of isoflavones or other phytoestrogens on OAB symptoms is sparse [44]. Animal studies have shown potentially beneficial effects on the bladder after intake of a soy-enriched diet [45,46]. In addition, Turgut et al. [47] found that isoflavone genistein reduced the expression of muscarinic receptors M2 and M3 on the bladder wall in ovariectomized rats, together with a reduction in unfavorable morphological changes connected to OAB, including decreased connective tissue and fibrosis. Based on these findings, they suggested that isoflavones may help to relax the bladder muscle and thereby improve the symptoms of OAB. Only a few clinical studies have been conducted on isoflavone-rich products, although with conflicting results, and most of them focus on urinary incontinence symptoms [48,49,50,51,52,53]. For instance, a randomized, cross-over trial testing a soy-rich diet on urogenital symptoms found no changes in urge urinary incontinence and vaginal dryness after 12 weeks [51], whereas other studies investigating soy/pumpkin seed extract intake showed reduced OAB symptoms [52,53]. Notably, pumpkin seeds contain lignans (another class of phytoestrogens) and have been suggested to reduce OAB symptoms [48,54]. Thus, it is not clarified whether isoflavones alone can have a beneficial effect on OAB symptoms. 

This study aimed to investigate the effect of isoflavones on OAB symptoms in postmenopausal women. Isoflavones were administered using a biofermented red clover extract (RCE) with high concentrations of the aglycone forms of the isoflavones formononetin and biochanin A.

## 2. Materials and Methods

### 2.1. Study Design

This study focusing on OAB symptoms was part of a three-month randomized, double-blinded, placebo-controlled trial (ClinicalTrials.gov NCT05013593) investigating RCE as a treatment for postmenopausal women with bladder symptoms. The primary outcome of the trial was changes in the urinary and vaginal microbiota composition, which will be reported elsewhere. In this current article, secondary outcome measures of OAB symptoms are reported. This includes changes in OAB symptom severity and incontinence assessed by two International Consultation on Incontinence Questionnaire (ICIQ) questionnaires—Overactive Bladder (ICIQ-OAB) [55] and Urinary Incontinence Short Form (ICIQ-UI-SF) [56,57]—and using a fluid intake and voiding diary.

**Figure 1:**
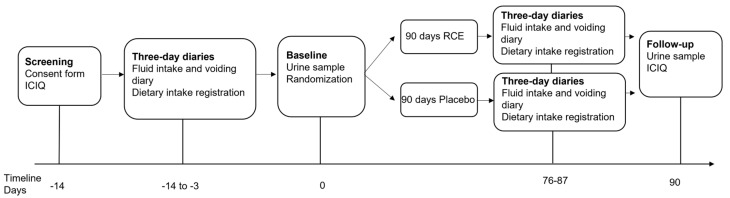
A schematic representation of the study design. Screening was scheduled approximately two weeks before the baseline visit, at which time, if the women decided to participate, they were asked to answer the ICIQ questionnaires to ensure eligibility. Before baseline and follow-up visits, the women were asked to fill out diaries. On the visit days (day 0 baseline and day 90 follow-up), a catheterized urine sample was collected, and questionnaires were filled in. At the baseline visit, the women were randomized and allocated to receive either the RCE or the placebo formulation. ICIQ: International Consultation on Incontinence Questionnaire; RCE: red clover extract.

The timeline of the study is illustrated in Figure 1. At screening, women were assessed for eligibility using the ICIQ questionnaires. Prior to baseline and follow-up visits, the women were asked to complete study diaries to measure their voiding habits and diet. The women were allocated to treatment groups based on randomization at the baseline visit, at which time a catheterized urine sample for culturing was collected. At the follow-up visit, the ICIQ questionnaires and a urine sample were collected again. 

#### Randomization and Double Blinding

The women were randomized to either RCE or placebo with a 1:1 allocation ratio between groups using the data management program Research Electronic Data Capture (REDCap) [58] hosted by the North Denmark Region. A blinded REDCap administrator generated the random allocation sequence. The women were enrolled and assigned an intervention by the research team. Both RCE and placebo were packed in identical sealed cardboard two-liter boxes and marked with a code (A or B) corresponding to the groups. All participants and the research team were blinded to the content of the boxes. The actual content of the boxes was revealed after study completion by an independent third party. 

### 2.2. Study Population

#### 2.2.1. Sample Size

We aimed for the inclusion of 50 women in each randomization arm. This was based on a previous study on RCE in the treatment of osteopenic postmenopausal women, where treatment effects were observed in a similar group of women [41]. 

#### 2.2.2. Participants

Participants were recruited through advertisements (posters, social media, and personal electronic mail from the public sector) from the community in Northern Denmark, at the North Denmark Regional Hospital, and Aalborg University Hospital. The first woman was enrolled in September 2019, and the last was enrolled in October 2021. Eligible participants were postmenopausal women with OAB with a minimum of five years since their last menstruation. Exclusion criteria were the use of systemic or local hormonal replacement therapy; intake of RCE or other isoflavone-rich supplements; use of prebiotic and/or probiotic supplements or receiving any antibiotics within three months prior to inclusion; recurrent urinary tract infections (defined as ≥2 infections in the last six months or ≥3 infections during the last year); current urinary tract infection (defined as a positive urine culture together with symptoms of cystitis, including dysuria, suprapubic tenderness/pain, hematuria, frequent urination, and urgency); and current or prior history of breast-, ovary-, or endometrial cancer and/or cancer of the digestive and/or urinary tract. Moreover, women who had used a levonorgestrel-releasing intrauterine device within the last five years and/or had a hysterectomy before the cessation of menstrual periods and were younger than 60 years were also excluded. 

### 2.3. Questionnaires and Data Acquisition

#### 2.3.1. Demographic and Clinical Data

At the baseline visit, health-demographic information (such as age, body mass index (BMI), smoking status, treatment, self-reported past medical/disease history, etc.) was collected using a structured questionnaire survey.

#### 2.3.2. ICIQ-OAB Questionnaire

OAB symptoms were assessed using a modified Danish translation of the ICIQ-OAB [55] (see Supplemental File S1), covering OAB symptoms over the past four weeks. The Danish ICIQ-OAB, generally used by urogynecologists in Denmark, includes four items on the frequency of micturition, nocturia, urgency, and urge urinary incontinence. A total ICIQ-OAB score ranging from 0 to 16 points was calculated, with higher scores indicating greater severity. Moreover, each item has an accompanying Numeric Rating Scale (NRS) to monitor participant-reported bother from 0 (not at all) to 10 (a great deal). OAB status for participant eligibility in the study was based on the women’s response to the ICIQ-OAB. We included women who replied “most of the time” or “all of the time” to question 5a, “Do you have to rush to the toilet to urinate?”. If they replied “sometimes” in item 5a, they had to respond “every second hour” or “every hour” to question 3a, “How often do you pass urine during the day?”. Furthermore, OAB type (dry or wet) was assessed. Wet OAB includes the symptom of urge urinary incontinence, whereas dry OAB does not. Urge urinary incontinence is the complaint of involuntary loss of urine associated with urgency (sudden desire to void). Women were not excluded if they had concomitant stress urinary incontinence (involuntary loss of urine on effort or physical exertion or on sneezing or coughing). 

#### 2.3.3. ICIQ-UI-SF Questionnaire

Overall severity and bother of urinary incontinence, together with the type of incontinence, were evaluated using the Danish version of the ICIQ-UI-SF questionnaire [56,57]. The questionnaire consists of four items regarding the frequency of urinary incontinence, severity, quality of life, and type of incontinence during the past four weeks. The responses to the first three items were used to obtain a total score ranging from 0 to 21 points, with higher scores indicating greater severity of urinary incontinence. The response to the last item was used to determine whether the women had urge urinary incontinence alone or urge urinary incontinence and stress urinary incontinence combined.

#### 2.3.4. Fluid Intake and Voiding Diary

To objectively evaluate the frequency of urination, bladder volume, and incontinence episodes, the women were asked to complete a three-day fluid intake and voiding diary, following the considerations of the International Consultation on Incontinence [2]. The diary consisted of consecutive days (if possible), including one weekend day. Information on measured fluid intake during the three days was included. If bedtime and waking were not indicated in the diary, these were defined as 12 p.m. (midnight) and 7 a.m., respectively. Parameters calculated from the diary, as an average of three days, were: total volume of fluid intake per 24 h, total volume of fluid intake at night, 24-h urine volume (summation of all urine volumes voided per 24 h), nocturnal urine volume (total urine volume at night, e.g., cumulative urine volume from voids after going to bed, including the first void at the time of waking, here given as the average of two days only), average voided volume, 24-h frequency (total number of daytime voids and episodes of nocturia), nocturnal frequency, daytime urinary frequency, 24-h incontinence episodes, incontinence episodes at night, 24-h maximum volume (maximum single void per 24 h), and daytime maximum volume (maximum single void during daytime). All data entries from the fluid intake and voiding diary were double-checked by a member of the research team.

#### 2.3.5. Diet Intake Registration

The women were also asked to complete a three-day food diary to monitor habitual diets, including the consumption of isoflavone-rich food. Food items were either weighed individually or given in portions. Each food diary was typed into an online diet and nutrition calculation program (VITAKOST ApS, Kolding, Denmark) by a single person from the research team. A second team member performed calculations on a subset of randomly selected samples (*n* = 20) to test for the reproducibility of analyzing the diet registration. There were no significant differences overall between the data from the two analyzers (Supplemental Figure S1). All data entries from the food diary were double-checked by a member of the research team. 

### 2.4. Standard Urine Culture

At the baseline and follow-up visits, a urine sample was collected using sterile intermittent catheterization to assess if a current urinary tract infection was present. Ten mL of urine was collected in a Urine Monovette with boric acid (Saarstedt, Germany) and analyzed by standard urine culturing.

### 2.5. Red Clover Extract and Placebo Formulations

The RCE and placebo formulations were produced by Herrens Mark ApS (Odense, Denmark). A heterogeneous culture (proprietary) of bacteria was used to facilitate the cold fermentation of pressed red clover to improve the bioavailability of the isoflavones. The isoflavone content in the RCE was determined as previously described [41], but with some modifications to the protocol. Isoflavones were detected using high-performance liquid chromatography (Agilent, Santa Clara, United States: pump G1311B, autosampler G1329B, column oven G1316A) with diode-array detection (Agilent G1315C). Four standards of aglycone isoflavones (genistein, daidzein, formononetin, and biochanin A) obtained from Sigma Aldrich (Brøndby, Denmark) and one glycoside derivative (Ononin, USP standard) were used for quantification of the isoflavones. The RCE was diluted in methanol, 10 mg to 50 mL methanol, before analysis (for contents, see Supplemental Table S1). 

For the study, the two treatments were prepared using RCE from a single batch or water with the addition of stevia (1.2 g/L), natural sugar-free flavor (1.5 g/L orange and 1 g/L raspberry), and brown food coloring (ammoniated caramel) (10 g/L). The RCE and placebo were packed in identical, sealed cardboard two-liter boxes. Participants consumed 35 mL of RCE or placebo twice a day: self-dosing in the morning and evening with a meal, corresponding to a total daily intake of 58.38 mg isoflavones (58.1 mg aglycones/d) for the RCE group and 0 mg isoflavones for the placebo group.

### 2.6. Compliance and Adverse Events

Compliance was calculated based on self-reported diaries. The women were instructed to register in the diary every time they consumed the treatment, by which the percentage of missing intakes could be calculated. This was subtracted from 100% to calculate compliance. Adverse events were monitored every month during the trial period by a telephone call from a research team representative and at the final follow-up visit.

### 2.7. Statistical Analysis

Data were analyzed using R Statistical Software (v4.1.3; R Core Team 2022, https://www.r-project.org/, accessed on 10 March 2022) through the Rstudio IDE (https://posit.co/, accessed on 10 March 2022). Mean and median values are stated as mean ± SD and median (Q1–Q3), respectively. For continuous data (age, BMI, etc.), distribution and variance were assessed using the Shapiro-Wilks test and Bartlett’s test, respectively. Normally distributed data with equal variance were compared using the Student’s *t*-test for independent data (comparison of baseline or follow-up data between groups) and the paired *t*-test for dependent data (comparison from baseline to follow-up). Non-normal distributed data were analyzed using the Mann-Whitney U test for independent data and the Wilcoxon test for dependent data. Categorical data were analyzed using the Chi-squared test, Fisher’s exact test, or the z-test. A *p*-value of < 0.05 was considered significant for all statistical tests. 

### 2.8. Ethics

The trial was conducted according to the principles expressed in the Declaration of Helsinki and was executed at the North Denmark Regional Hospital. The study was approved by The North Denmark Region Committee on Health Research Ethics (N-20190028). The categories of personal data collected in the project were registered in the processing activities of research in the North Denmark Region in compliance with EU GDPR Article 30. Oral and written informed consent was obtained from all participants.

## 3. Results

### 3.1. Study Participants

**Figure 2:**
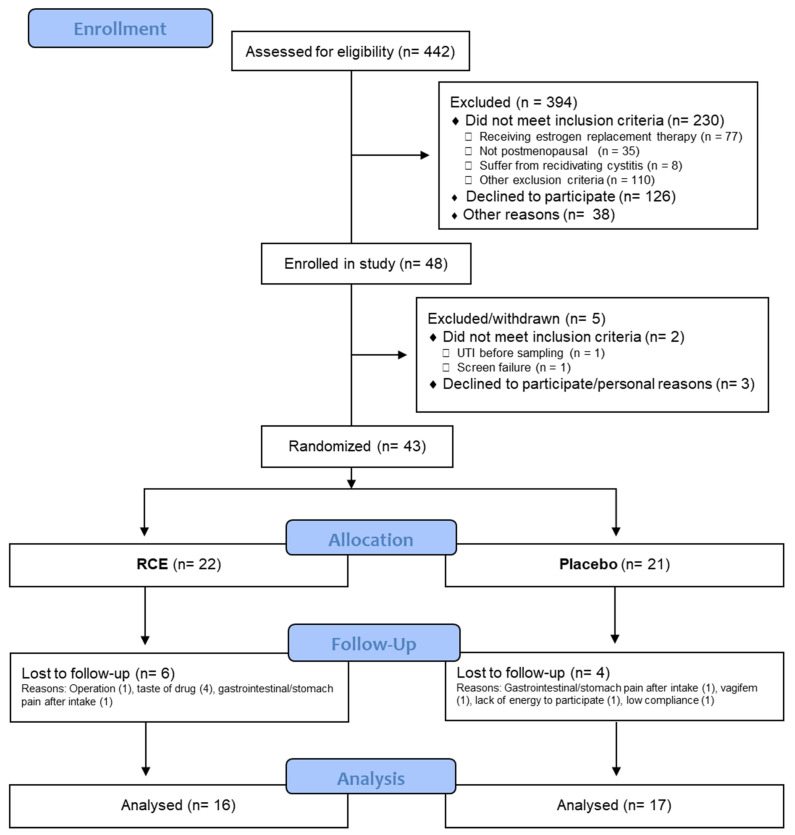
Consort participant flowchart of participation through the present study. UTI: urinary tract infection; RCE: red clover extract.

A total of 48 women were enrolled in the study (Figure 2). Five were subsequently excluded before randomization, and an additional 10 were lost by follow-up. This resulted in 33 women being included in the final data analysis. 

### 3.2. Baseline Characteristics

Table 1 shows the baseline characteristics of the 33 women included in the data analysis, with 16 in the RCE group (mean age 62.9 ± 6.1 years) and 17 in the placebo group (mean age 62.8 ± 6.5 years). There were no significant differences in any of the demographic and clinical data between women receiving RCE versus placebo (Table 1). No differences were observed between groups regarding OAB subtype or symptom severity. Several of the women with a wet OAB suffered from both urge and stress urinary incontinence (Table 1). Furthermore, women in the RCE group and placebo group had comparable parameters from the fluid intake and voiding diary at baseline (Supplemental Table S2). Based on the food diaries, no differences in diet composition were observed between the two groups (Supplemental Table S3). One woman in the RCE group was taking mirabegron (Betmiga) and was still included due to compliance with symptom criteria but terminated treatment during the study period. Furthermore, one woman in the RCE group was taking loop diuretics (Bumetanid, Burinex^®^,, Karo Pharma, Stockholm, Sweden). Due to an infection (location unknown) during the study period, two women received antibiotics.

### 3.3. ICIQ-OAB Score

**Figure 3:**
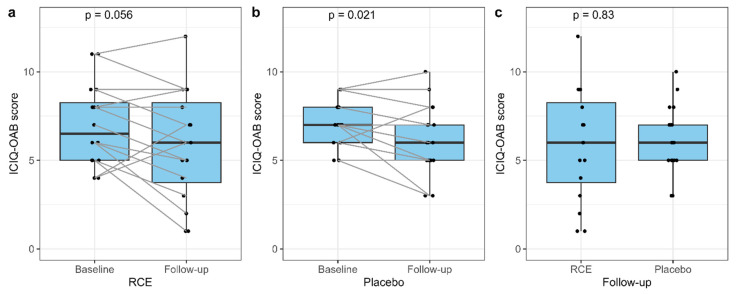
International Consultation on Incontinence Questionnaire Overactive Bladder (ICIQ-OAB) score at baseline and follow-up for RCE (**a**), placebo (**b**), and comparison at follow-up (**c**). The comparison from baseline to follow-up was analyzed using the paired samples Wilcoxon test. The comparison of RCE (red clover extract) and placebo at follow-up was analyzed using the Mann-Whitney U test.

A tendency was seen towards a decrease in the ICIQ-OAB score in the RCE group from baseline to follow-up (with a median score of 6.5 (5.0−8.25) at baseline versus 6.0 (3.75−8.25) at follow-up (*p* = 0.056)) (Figure 3a). In the placebo group, the median ICIQ-OAB score declined significantly from 7.0 (6.0−8.0) at baseline to 6.0 (5.0−7.0) at follow-up (*p* = 0.021) (Figure 3b). No significant difference in the ICIQ-OAB score between the two groups was found at follow-up (*p* = 0.83) (Figure 3c).

### 3.4. Individual ICIQ-OAB Items 

**Figure 4:**
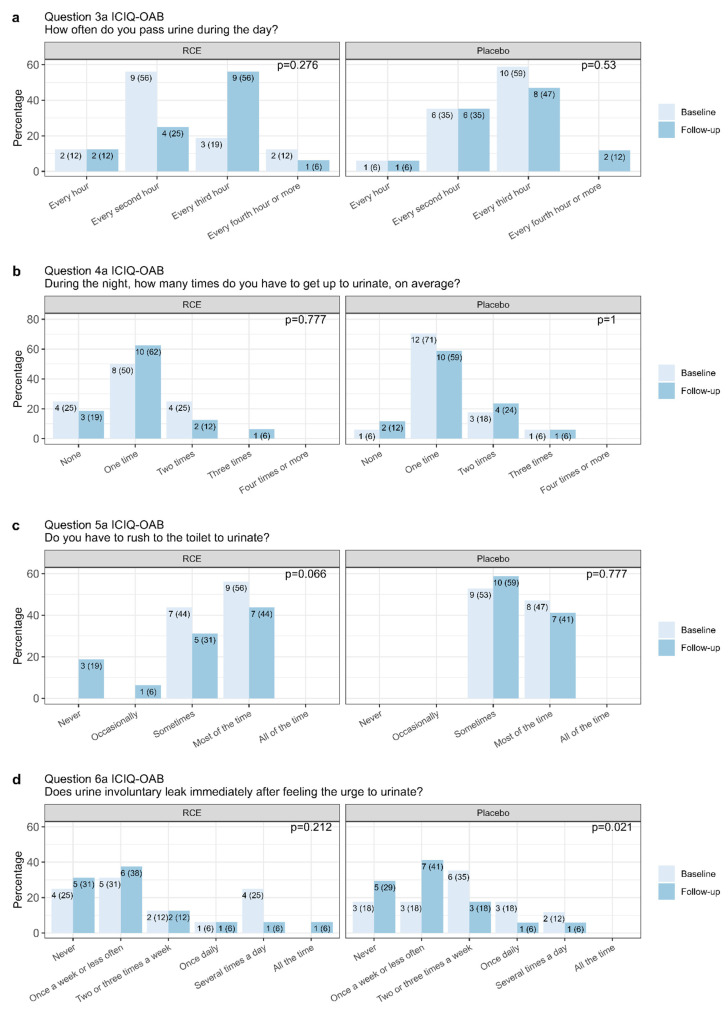
International Consultation on Incontinence Questionnaire Overactive Bladder (ICIQ-OAB) results from questions 3a (**a**), 4a (**b**), 5a (**c**), and 6a (**d**). The count (%) of answers within each question is stated in the bar chart and grouped for the RCE (red clover extract) or placebo group at baseline and three-month follow-up. Statistical comparison of baseline and follow-up using the paired samples Wilcoxon test.

When looking at individual items in the ICIQ-OAB questionnaire, we found no differences in urinary frequency (question 3a) or nocturia (question 4a) for either the RCE or placebo group (Figure 4a,b). However, a tendency towards a reduction in urgency symptoms (question 5a) in the RCE group was observed at follow-up compared to the placebo group (Figure 4c). The shift was, however, not significant. There appeared to be a significant improvement in the number of daily urge urinary incontinence episodes (question 6a) for the placebo group (*p* = 0.021), which was not observed in the RCE group (*p* = 0.212) (Figure 4d). 

### 3.5. Patient-Reported Symptom Bother (NRS Values) for Urinary Frequency, Nocturia, Urgency, and Urge Urinary Incontinence from the ICIQ-OAB Questionnaire

In the RCE group, a reduction was detected in the bother of urinary urgency on an NRS (0–10) from baseline to follow-up (*p* = 0.033, Table 2). In contrast, no difference was found between the RCE group and placebo group at follow-up (*p* = 0.44, Table 2). Furthermore, no other differences between the two groups concerning patient-reported symptom bother were observed (Table 2). 

### 3.6. The ICIQ-UI Score

**Figure 5:**
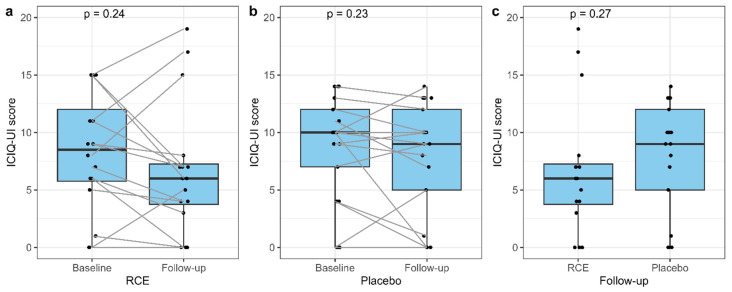
International Consultation on Incontinence Questionnaire Urinary Incontinence (ICIQ-UI) score at baseline and follow-up for RCE (**a**), placebo (**b**), and comparison at follow-up (**c**). The comparison from baseline to follow-up was analyzed using the paired samples Wilcoxon test. The comparison of RCE (red clover extract) and placebo at follow-up was analyzed using the Mann-Whitney U test.

No significant difference in the ICIQ-UI score was found between baseline and follow-up in either the RCE group or placebo group (Figure 5a,b), indicating no improvements in the severity of urinary incontinence symptoms after intervention. Similarly, no significant difference was found at follow-up between the two groups (median score 6.0 (3.75−7.25) for the RCE group and 9.0 (5.0−12.0) for the placebo group, *p* = 0.27) (Figure 5c). 

### 3.7. Fluid Intake and Voiding Diary

Table 3 shows the results from the fluid intake and voiding diary. No significant differences from baseline to follow-up in any of the investigated parameters were found in the RCE group (Table 3). However, a significant reduction in the number of daily micturitions (*p* = 0.01) and the 24-h frequency (*p* = 0.012) was found in the placebo group (Table 3).

### 3.8. Compliance and Adverse Events

The median duration of the treatment in the trial was 99.5 (91–107) days, and the median compliance was 96% (90.9–98.5%). The treatments were generally well tolerated. Four women taking RCE had difficulties with the taste, and two women, one in each treatment group, experienced stomach pain after intake; these women all dropped out of the study.

## 4. Discussion

The present study showed no clear improvements in the majority of OAB symptom parameters in postmenopausal women suffering from OAB following treatment with fermented RCE compared to a placebo. OAB is a complex syndrome, and its pathophysiology is not fully understood [59]. OAB symptoms can arise due to various factors, such as recurrent urinary tract infections, bladder cancer, and neurological disorders, among others. Additionally, the symptoms, including severity and bother, are highly individualized and can be influenced by factors such as fluid intake, quality of sleep, and physical challenges in reaching the toilet on time. As a result, women experience a diverse array of symptoms [59]. This complexity affects the assessment of symptom relief following treatment, and therefore several measures have been developed to cover this challenge, including several patient questionnaires [60,61,62,63] and bladder diaries [64]. In this study, an assessment of OAB symptoms was performed using both questionnaires and a fluid intake and voiding diary to reflect this complexity.

By evaluating the ICIQ-OAB questionnaire, we saw tendencies, though not statistically significant, towards a reduced ICIQ-OAB score (*p* = 0.056) in women receiving RCE. The same tendency was observed towards reduced symptoms of urgency (*p* = 0.066), indicating that some of the women in our study experienced relief related to urgency. In contrast, no improvements regarding episodes of urge urinary incontinence, urinary frequency, or nocturia were found. However, we observed a decrease in the patient-reported bother of urinary urgency (*p* = 0.033) after treatment with RCE. Importantly, we found significant improvements related to the total ICIQ-OAB score and episodes of urge urinary incontinence in the placebo group, indicating a placebo effect. The discovery of a placebo effect is common, and a decrease in symptom scores in other studies investigating treatment of OAB using the ICIQ-OAB score has been found [63].

The overall burden and bother of urinary incontinence evaluated by the ICIQ-UI-SF were not improved in either of the groups. A reason for this may be explained by the fact that a large part of the women in our study suffered from stress urinary incontinence (50% in the RCE group and almost 65% in the placebo group), which we do not expect the RCE to be effective against.

No improvements in urinary frequency or other voiding parameters from the fluid intake and voiding diary were found in women taking RCE. However, women in the placebo group reported a significantly lower number of micturitions. A similar level of improvement (e.g., reduction of around one void/day) has been observed in the control group in other studies aiming to control OAB symptoms [53,65]. This placebo effect is believed to be a result of participating in a study and/or drawing attention to voiding habits. It may also be explained by a decrease (though not statistically significant) in fluid intake during the trial. Furthermore, the women in our study generally had a higher average voided volume and lower urinary frequency compared with other studies [61,65,66]. Thus, our participants had less severe OAB symptoms. 

Overall, we observe an agreement between the responses to the ICIQ-OAB items and parameters reported in the fluid intake and voiding diary. For example, although not statistically significant, a slight increase in urine volume was observed in the RCE group. This change may indicate a tendency toward decreased urgency, as demonstrated in the ICIQ-OAB responses. In contrast, a few observations from the ICIQ-OAB and diary do not agree. In the diary, we find a significant decrease in the numbers of micturitions for the placebo group, but this is not demonstrated in the ICIQ-OAB item 3a. 

Other clinical studies looking at the effect of isoflavones on symptoms suggestive of OAB are sparse and mainly focus on urinary incontinence or vaginal symptoms. Yet, our results are consistent with previous findings where intake of isoflavones was not associated with improvements in urinary incontinence, frequency, or urgency [48,50,51,67,68]. However, a direct comparison between studies is challenged by methodological heterogeneity and differences in outcome measures, and the assessment of symptoms suggestive of OAB has often been a matter of secondary importance. Furthermore, none of the studies have focused on women with OAB at the time of inclusion. Instead, they include women more broadly with urogenital problems—not just bladder symptoms but also vaginal dryness and sexual problems [51,67]. Other studies include participants more generally based on the presence of menopausal symptoms according to the Menopause Rating Scale [68] or healthy women with no incontinence [50]. This makes it difficult to compare with our study, and direct conclusions related to OAB are hard to draw. Furthermore, the amount and composition of isoflavones investigated vary between studies. In the study by Waetjen et al. [50], isoflavone intake is estimated from the diet of the participants. Contrary to this, the study by Juliato et al. [49] found that current or previous use of soy products to treat menopausal symptoms was associated with a lower prevalence of urinary incontinence. Still, this study was a cross-sectional study, and the amount and preparation of soy products were not indicated.

There may be multiple reasons why we only detected a limited improvement in bladder symptoms. First, it may be attributed to insufficient ER expression in the bladder. Earlier studies have shown that ER-β is the dominant ER in the bladder [26,69,70] and that isoflavones selectively target ER-β [24,71], leading to our hypothesis that isoflavones may be able to target the bladder and compensate for age-related estrogen loss. However, it is unknown if the expression of ERs changes with OAB symptom progression and/or estrogen status. One study found that the expression of ER-β was either decreased or absent in the vaginal wall in postmenopausal women compared to premenopausal women [72]. In contrast, ER-α was expressed equally in premenopausal and postmenopausal women [72]. In our study, we have not determined ER expression levels and cannot conclude if this had any influence on our results. Second, RCE is ingested orally. Compared to exogenous estrogen therapy, systemic treatment with estrogen also fails to improve OAB symptoms [15,16,17,18,19]. Vaginal estrogen therapy, on the other hand, has shown beneficial improvements with reduced urinary frequency, urgency perception, nocturia, and vaginal dryness [10,11,12,13,14]. However, some of these studies lack a placebo control group [13,14]. Local vaginal treatment with isoflavones could be a possible treatment opportunity. Lima et al. [73] found that an isoflavone vaginal gel relieves vaginal dryness and dyspareunia symptoms in postmenopausal women, similar to a conjugated equine estrogen gel. These treatments were superior to the placebo gel [73]. Future investigations on the topic may be interesting. 

### Strengths and Limitations

Some limitations should be considered. OAB symptom complexity can be influenced by many factors, such as the type of fluid intake. However, we did not have information about the participants’ consumption of different types of fluids, e.g., caffeine-containing drinks, which are known to affect voiding habits. The sample size in our study was small, making it difficult to obtain statistical conclusions and account for heterogeneity among participants. We formerly aimed for 100 participants, but due to problems finding women meeting study criteria, we enrolled a lower number. Additionally, compliance was not based on measuring the residual volume of the RCE and placebo. The use of a randomized, double-blinded, placebo-controlled study design entails both limitations and strengths for the study. It results in a lower number of women included in each treatment group; however, it also enables the investigation of a placebo effect. Another strength is that we evaluated OAB symptom effects using both ICIQ questionnaires in combination with a fluid intake and voiding diary.

## 5. Conclusions

The present study has not been able to demonstrate the effect of systemic treatment with isoflavones on the relief of OAB symptoms in postmenopausal women. Larger studies are needed to unravel the possible role of isoflavones in female bladder disorders. In addition, better in-depth knowledge of the mechanisms that isoflavones may have on ER binding in the bladder is warranted.

## Figures and Tables

**Table 1:** Baseline health-demographic characteristics of the study participants according to randomization group.

Parameters	Total (*n* = 33)	RCE (*n* = 16)	Placebo (*n* = 17)	*p*
Age (years), mean ± SD	62.8 ± 6.2	62.9 ± 6.1	62.8 ± 6.5	0.938 ^a^
Body mass index (kg/m^2^) *, median (QR1–QR3)	25.0 (22.0–30.9)	25.6 (20.7–31.0)	24.4 (22.7–28.6)	0.909 ^b^
Smoking status, *n* (%) - Never smoked - Former smoker - Smoker	15 (45.5) 11 (33.3) 7 (21.2)	8 (50.0) 5 (31.2) 3 (18.8)	7 (41.2) 6 (35.3) 4 (23.5)	0.874 ^c^
Number of cigarettes/day, mean ± SD - Former smoker - Smoker	12.9 ± 6.8 6.0 ± 1.7	12.6 ± 8.1 6.3 ± 1.2	13.2 ± 6.3 5.8 ± 2.2	0.902 ^a^ 0.672 ^a^
Number of births, median (QR1–QR3)	2.0 (2.0–2.0)	2.0 (2.0–2.25)	2.0 (2.0–2.0)	0.704 ^c^
Number of cesareans ^◊^, median (QR1–QR3)	0 (0–0)	0 (0–0)	0 (0–0)	0.570 ^c^
OAB type, *n* (%) - Wet - Dry	18 (54.5) 15 (45.5)	7 (43.8) 9 (56.2)	11 (64.7) 6 (35.3)	0.391 ^c^
Urinary incontinence type, *n* (%) - Urge - Urge and stress - Stress ^□^ - No urinary incontinence	4 (12.1) 14 (42.4) 5 (15.1) 10 (30.3)	2 (12.5) 5 (31.2) 3 (18.8) 6 (37.5)	2 (11.8) 9 (52.9) 2 (11.8) 4 (23.5)	0.684 ^d^
ICIQ-UI score (0–21), median (QR1–QR3)	9.0 (6.0–12.0)	8.5 (5.8–12.0)	10.0 (7.0–12.0)	0.800 ^b^
ICIQ-OAB score (0–16), median (QR1–QR3)	7.0 (6.0–8.0)	6.5 (5.0–8.25)	7.0 (6.0–8.0)	0.728 ^b^
Selected diseases ⁰, self-reported, *n* (%) Urogenital - Lichen sclerosus - Prolaps Endocrine - Diabetes mellitus 2 - Hypothyroidism	1 (3.1) 1 (3.1) 1 (3.1) 2 (6.3)	1 (6.25) 1 (6.25) 1 (6.25) 1 (6.25)	0 (0.0) 0 (0.0) 0 (0.0) 1 (6.25)	1 1 1 1

Percentages are calculated based on either the total or within each treatment group. ^a^ *t*-test for unpaired means comparisons; ^b^ Mann-Whitney U test; ^c^ Chi-squared test; ^d^ Fisher’s exact test. * Body mass index: one woman in the placebo group did not report weight (*n* = 16). ^◊^ One woman in the RCE group has had two cesareans, whereas two in the RCE group and placebo group have had a single cesarean. ^□^ Stress urinary incontinence type is dry OAB with stress urinary incontinence. ⁰ Other diseases are self-reported. Only selected diseases are shown (for the full list, see Supplemental Table S4). One woman in the placebo group did not report current or previous other diseases (*n* = 16). Comparison of proportions by z-test. RCE: red clover extract; OAB: overactive bladder.

**Table 2:** Comparison of patient-reported symptom bother (NRS values) from the ICIQ-OAB questionnaire.

OAB NRS Values	RCE (*n* = 16)			Placebo (*n* = 17)			Difference between Groups
	Baseline	Follow-Up	*p*	Baseline	Follow-Up	*p*	*p* ^a^
Urinary frequency bother (0–10)	5.0 (1.5–6.3)	1.0 (0.0–8.3)	0.263	5.0 (4.0–6.0)	5.0 (3.0–6.0)	0.245	0.19
Nocturia bother (0–10)	3.0 (0.8–7.0)	2.0 (0.0–4.3)	0.248	5.0 (1.0–6.0)	5.0 (2.0–6.0)	0.633	0.11
Urgency bother (0–10)	7.0 (4.0–8.3)	4.5 (0.8–8.0)	0.033	7.0 (5.0–7.0)	5.0 (4.0–7.0)	0.138	0.44
Urge urinary incontinence bother (0–10)	4.5 (0.0–7.5)	3.5 (0.0–8.0)	0.501	6.0 (3.0–8.0)	5.0 (1.0–7.0)	0.403	0.7

NRS values are median (QR1−QR3). The NRS to monitor the bother of the symptom ranges from 0 (not at all) to 10 (a great deal). The comparison from baseline to follow-up was analyzed using the paired samples Wilcoxon test. ^a^ The comparison of RCE (red clover extract) and placebo at follow-up was analyzed using the Mann-Whitney U test.

**Table 3:** Fluid intake and urinary voiding information as reported by the women in the study diary in accordance with trial randomization status.

Fluid Intake and Voiding Diary	RCE (*n* = 15 *)			Placebo (*n* = 17)			Difference between Groups
	Baseline	Follow-Up	*p*	Baseline	Follow-Up	*p*	*p* ^a^
Fluid intake 24-h median (QR1–QR3)	1897 (1498–2022)	1750 (1438–2087)	0.359	1883 (1617–2033)	1513 (1417–1967)	0.145	0.929
Fluid intake night median (QR1–QR3)	0 (0–62.5)	33.3 (0–100)	0.824	0 (0–0)	0 (0–0)	0.834	0.106
24-h urine volume mean ± SD	1768 ± 502	1820 ±449	0.738	1780 ± 503	1684 ± 465	0.268	0.408
Nocturnal urine volume median (QR1–QR3)	410 (274–612)	400 (282–715)	0.890	450 (335–550)	425 (400–630)	0.636	0.734
Average voided volume mean ± SD	198 ± 71.2	217 ± 75.7	0.072	206 ± 62.7	221 ± 53.6	0.237	0.875
24-h frequency mean ± SD median (QR1–QR3)	9.33 ± 1.98 8.67 (8.17–11.0)	8.93 ± 2.59 8.67 (7.5–11.3)	0.422	8.94 ± 2.23 8.33 (7.67–9.33)	7.90 ± 2.43 7 (6.33–8.33)	0.012	0.108
Nocturnal frequency median (QR1–QR3)	1.67 (0.67–2.33)	1 (0.67–1.33)	0.349	0.67 (0.33–1.33)	1 (0.33–1.33)	0.861	0.659
Daytime urinary frequency mean ± SD median (QR1–QR3)	7.89 ± 1.88 7.67 (6.83–9.17)	7.69 ± 2.05 7.67 (6.83–9.17)	0.578	7.98 ± 1.68 7.67 (7–9.33)	6.98 ± 2.14 6 (5.67–7.67)	0.009	0.173
24-h incontinence episodes ** median (QR1–QR3)	0.33 (0.0–1.33)	0.0 (0.0–0.67)	0.612	0.67 (0.0–2.0)	0.0 (0.0–1.0)	0.089	0.627
Incontinence episodes night ** mean ± SD median (QR1–QR3)	0.17 ± 0.64 0 (0–0)	0.10 ± 0.21 0 (0–0)	0.854	0.15 ± 0.42 0 (0–0)	0.09 0 (0–0)	0.391	0.229
24-h max volume median (QR1–QR3)	500 (300–585)	460 (315–575)	0.72	460 (350–500)	500 (350–600)	0.051	0.471
Daytime max volume median (QR1–QR3)	440 (285–500)	450 (300–550)	0.345	460 (300–500)	400 (320–500)	0.955	0.995

Mean ± SD are stated for normally distributed data, whereas median (QR1–QR3) values are stated for not normally distributed data. All volumes are in mL. * One woman did not complete the fluid intake and voiding diary at follow-up and was therefore excluded from the analysis. ** Two women did not report incontinence episodes and were therefore excluded from the analysis of incontinence. The comparison from baseline to follow-up was analyzed using the paired *t*-test for normally distributed data and the paired samples Wilcoxon test for non-normal distributed data. ^a^ The comparison of RCE (red clover extract) and placebo at follow-up was analyzed using the Student’s *t*-test for normally distributed data and the Mann-Whitney U test for non-normal distributed data.

## Data Availability

All data generated or analyzed in the study are included in this published article and its supplementary materials.

## Supplementary Materials

The following supporting information can be downloaded at: https://www.mdpi.com/article/10.3390/nu15194165/s1, File S1: ICIQ-OAB questionnaire used in this study. Figure S1: Food diary. A comparison of a subset of randomly selected participants (*n* = 20) to test for reproducibility between two different analyzers. Table S1: Red clover extract isoflavone composition and content; Table S2: Fluid intake and bladder diary characteristics at baseline; Table S3: Dietary intake registration; Table S4: Self-reported diseases by participants.
