# Supplementary material for: Use of Fermented Red Clover Isoflavones in the Treatment of Overactive Bladder in Postmenopausal Women: A Randomized, Double-Blinded, Placebo-Controlled Trial
Source: Nutrients. 2023 Sep 27;15(19):4165. doi: 10.3390/nu15194165 (PMC10574253; doi:10.3390/nu15194165)
Supplement: Supplementary file 1 [file nutrients-15-04165-s001.zip › nutrients-2602983-supplementary file S1.pdf]

Use of fermented red clover isoflavones in the treatment of overactive bladder in postmenopausal women: A randomized, double-blinded, placebo-controlled trial

Annemarie B. Villadsen [1,2], Julie N. Holm-Jacobsen [1], Bala K. Prabhala [3], Caspar Bundgaard-Nielsen [1,2], Pam Huntjens [1], Jette B. Kornum [4], Karin Glavind [5], Peter D. C. Leutscher [1,2], Lars P. Christensen [3], Per B. Jeppesen [6], Suzette Sørensen [1,2,7], Louise T. S. Arenholt [1,2,8,*]

Supplemental file S1: ICIQ-OAB used in the study

Participant ID: ________________

Overactive bladder ICIQ-OAB 08/04

Many people experience urinary symptoms some of the time. We are trying to find out how many people experience urinary symptoms, and how much they bother them. We would be grateful if you could answer the following questions, thinking about how you have been, on average, over the PAST FOUR WEEKS.

1. Please write in your date of birth: ☐☐ ☐☐ ☐☐☐☐

Day Month Year

3a. *How often do you pass urine during the day?*

Every hour	☐	3
Every second hour	☐	2
Every third hour	☐	1
Every fourth hour or more	☐	0

3. *How much does this bother you?*
Please ring a number between 0 (not at all) and 10 (a great deal)

0 1 2 3 4 5 6 7 8 9 10
not at all … **a great deal**

4a. *During the night, how many times do you have to get up to urinate, on average?*

None	☐	0
One time	☐	1
Two times	☐	2
Three times	☐	3
Four times or more	☐	4

4b. *How much does this bother you?*
Please ring a number between 0 (not at all) and 10 (a great deal)

0 1 2 3 4 5 6 7 8 9 10
not at all … **a great deal**

5a. *Do you have to rush to the toilet to urinate?*

Never	☐	0
Occasionally	☐	1
Sometimes	☐	2
Most of the time	☐	3
All of the time	☐	4

5b. *How much does this bother you?*
Please ring a number between 0 (not at all) and 10 (a great deal)

0 1 2 3 4 5 6 7 8 9 10
not at all **a great deal**

6a. *Does urine involuntary leak immediately after feeling the urge to urinate?*

Never	☐	0
Once a week or less often	☐	1
Two or three times a week	☐	2
Once daily	☐	3
Several times a day	☐	4
All the time	☐	5

6b. *How much does this bother you?*
Please ring a number between 0 (not at all) and 10 (a great deal)

0 1 2 3 4 5 6 7 8 9 10
not at all **a great deal**

Thank you very much for answering these questions.
