# Supplementary material for: Use of Fermented Red Clover Isoflavones in the Treatment of Overactive Bladder in Postmenopausal Women: A Randomized, Double-Blinded, Placebo-Controlled Trial
Source: Nutrients. 2023 Sep 27;15(19):4165. doi: 10.3390/nu15194165 (PMC10574253; doi:10.3390/nu15194165)
Supplement: Supplementary file 1 [file nutrients-15-04165-s001.zip › nutrients-2602983-supplementary.pdf]

Use of fermented red clover isoflavones in the treatment of overactive bladder in postmenopausal women: A randomized, double-blinded, placebo-controlled trial

Annemarie B. Villadsen [1,2], Julie N. Holm-Jacobsen [1], Bala K. Prabhala [3], Caspar Bundgaard-Nielsen [1,2], Pam Huntjens [1], Jette B. Kornum [4], Karin Glavind [5], Peter D. C. Leutscher [1,2], Lars P. Christensen [3], Per B. Jeppesen [6], Suzette Sørensen [1,2,7], Louise T. S. Arenholt [1,2,8,*]

Supplemental Figure S1: Food diary. Comparison of a subset of randomly selected participants (N=20) to test for reproducibility between two different analyzers.

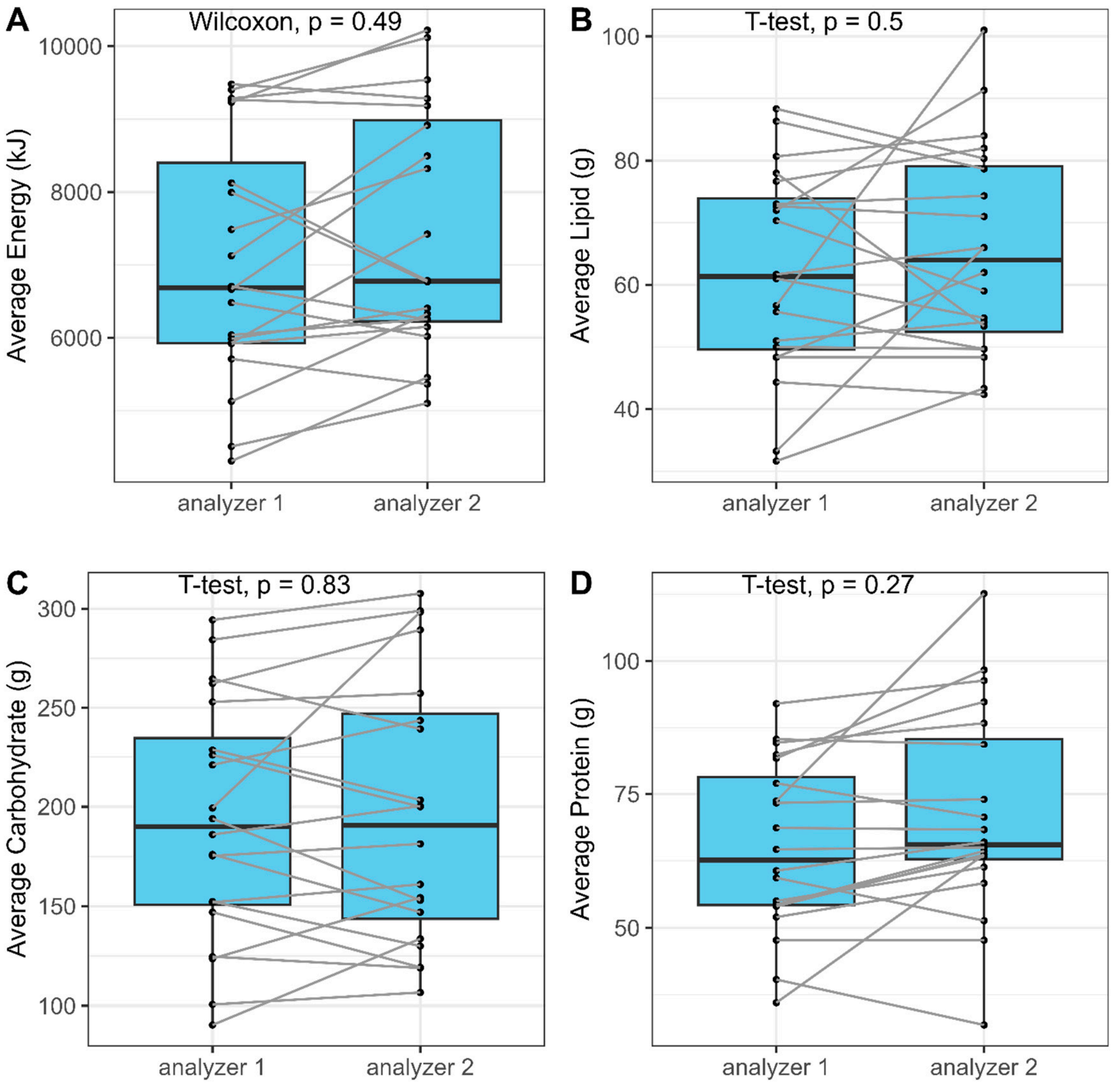

Supplemental Table S1: Red clover extract isoflavone composition and content

Isoflavone	mg/kg
**Formononetin**	541
**Biochanin A**	210
**Genistein**	65
**Daidzein**	14
**Ononin**	4
**Total isoflavone content**	834

Supplemental Table S2: Fluid intake and bladder diary characteristics at baseline

Parameters	RCE (n=16)	Placebo (n=17)	p-value
**Fluid intake 24-hour (mL)**<br>**Median (QR1-QR3)**	1897 (1498-2022)	1883 (1617-2033)	0.925
**Fluid intake night (mL)**<br>**Median (QR1-QR3)**	0 (0-62.5)	0 (0-0)	0.246
**24-hour urine volume (mL)**<br>**Mean ±SD**<br>**Median (QR1-QR3)**	1768 ±502<br>1557 (1416-2233)	1780 ±503<br>1853 (1343-2192)	0.948<br>1
**Nocturnal urine volume (mL)**<br>**Median (QR1-QR3)**	410 (274-612)	450 (335-550)	0.985
**Average voided volume (mL)**<br>**Mean ± SD**	198 ±71.2	206 ±62.7	0.727
**24-hour frequency**<br>**Median (QR1-QR3)**	8.67 (8.17-11.0)	8.33 (7.67-9.33)	0.472
**Nocturnal frequency**<br>**Median (QR1-QR3)**	1.67 (0.67-2.33)	0.67 (0.33-1.33)	0.119
**Daytime urinary frequency**<br>**Median (QR1-QR3)**	7.67 (6.83-9.17)	7.67 (7-9.33)	0.895
**24-hour incontinence episodes**<br>**Median (QR1-QR3)**	0.33 (0-1.33)	0.67 (0-2)	0.478
**Incontinence episodes night**<br>**Mean ± SD**<br>**Median (QR1-QR3)**	0.179 ± 0.647<br>0 (0-0)	0.157 ±0.427<br>0 (0-0)	0.524
**24-hour max volume (mL)**<br>**Median (QR1-QR3)**	500 (300-585)	460 (350-500)	0.649
**Daytime max volume (mL)**<br>**Median (QR1-QR3)**	440 (285-500)	460 (300-500)	0.506

Note. Mean ± SD are stated for normally distributed data whereas median (QR1-QR3) values are stated for not normally distributed data. All volumes are in mL. RCE: red clover extract.

Supplemental Table S3: Dietary intake registration

Dietary intake	RCE (n=16)$^{\Delta}$			Placebo (n=17)			Difference between groups
	Baseline	Follow-up	*p*	Baseline	Follow-up	*p*	p^a
**Energy, kJ**	7043 ±1352	7166 ±1784	0.764	6703 ±1132	6488 ±1163	0.281	0.21
**Lipid, g**	61.7 ±10.6	64.7 ±21.1	0.525	56.3 ±12.1	54.8 ±18.0	0.698	0.16
**Carbohydrate, g**	188 ±54.1	189 ±53.4	0.924	185 ±34.1	182 ±45.6	0.719	0.67
**Protein, g**	70.2 ±11.0	72.7 ±15.0	0.574	67.3 ±18.8	64.7 ±17.6	0.647	0.171

Note. Data are displayed as an average of the three days presented as means ±SD. Data do not include nutrient intake from supplements. $^{\Delta}$The food diary revealed that one woman consumed food that contained high levels of isoflavones, though in low amounts. [a] Comparison of RCE (red clover extract) with placebo at follow-up.

Supplemental Table S4: Self-reported diseases by participants.

Feature	RCE (n=16)	Placebo (n=16)	p-value
**Other diseases, n (%)**			
**Central nervous system**			
**- Stroke**	1 (6.25)	0 (0.0)	1
**- Migraine**	0 (0.0)	1 (6.25)	1
**- Parkinson's**	1 (6.25)	0 (0.0)	1
**Cardiovascular**			
**- Arteriosclerosis**	0 (0.0)	1 (6.25)	1
**- Hypertension**	3 (18.8)	3 (18.8)	1
**- Increased cholesterol**	6 (62.5)	3 (18.8)	0.432
**Lung / pulmonary**			
**- Chronic obstructive pulmonary disease**	1 (6.25)	0 (0.0)	1
**- Asthma**	1 (6.25)	1 (6.25)	1
**- Others**	1 (6.25)	0 (0.0)	1
**Gastrointestinal tract**			
**- Construction of cardiac sphincter**	1 (6.25)	0 (0.0)	1
**Musculoskeletal**			
**- Osteoarthritis**	7 (43.8)	3 (18.8)	0.253
**- Osteoporosis**	2 (12.5)	1 (6.25)	1
**- Back disease**	1 (6.25)	1 (6.25)	1
**- Hip fracture**	1 (6.25)	0 (0.0)	1
**Urogenital**			
**- Lichen sclerosus**	1 (6.25)	0 (0.0)	1
**- Prolaps**	1 (6.25)	0 (0.0)	1
**Endocrine**			
**- Diabetes mellitus 2**	1 (6.25)	0 (0.0)	1
**- Hypothyroidism**	1 (6.25)	1 (6.25)	1
**Cancer**			
**- Skin cancer**	1 (6.25)	0 (0.0)	1
**- Vulva cancer**	1 (6.25)	0 (0.0)	1

Note. Percentages are calculated based on each treatment group. One woman in the placebo group did not report current or previous other diseases (n=16). Comparison of proportions by z-test. RCE: Red clover extract
